# The distally anchored connective tissue graft platform for papilla enhancement: A case report

**DOI:** 10.1002/cap.10299

**Published:** 2024-06-10

**Authors:** Gonzalo Blasi, Lory Abrahamian, Alvaro Blasi

**Affiliations:** ^1^ Department of Periodontology School of Dentistry International University of Catalonia Barcelona Spain; ^2^ Division of Periodontics Department of Advanced Oral Sciences and Therapeutics University of Maryland, Baltimore School of Dentistry Baltimore Maryland USA; ^3^ Department of Restorative Dentistry School of Dentistry International University of Catalonia Barcelona Spain; ^4^ Department of Restorative Sciences Dental College of Georgia at Augusta University Augusta Georgia USA

**Keywords:** autografts, bone regeneration, gingival recession, interdental papilla, single‐tooth dental implant, tooth socket

## Abstract

**Background:**

As the need for using dental implants to replace single missing teeth grows, so does the demand for greater esthetic results. However, achieving complete interproximal papillae fill in single‐tooth implant restorations remains a challenge. The distally anchored connective tissue platform is a novel soft tissue augmentation technique that consists of harvesting an autogenous connective tissue graft from the palate, folding it, and positioning it at the level of the distal occlusal and buccal surfaces with the help of a distal sling suture to the adjacent distal tooth.

**Methods:**

This case report describes how a maxillary central incisor with compromised hard and soft tissues were replaced using a comprehensive treatment plan.

**Results:**

The clinical outcomes showed stable mucosal margin levels and complete papillae fill. The patient expressed satisfaction with the achieved results.

**Conclusions:**

The distally anchored connective tissue graft platform performed at the time of implant placement emerges as a viable and effective soft tissue augmentation technique that yields highly esthetic results.

**Key points:**

**Why is this case new information?**

- To the best of our knowledge, this is the first case report in the literature using the distally anchored connective tissue platform.

**What are the keys to successful management of this case?**

- Adequate diagnosis and decision‐making, resulting in a treatment plan focused on reconstructing both soft and hard tissues in a single‐tooth implant within the esthetic area, yield favorable clinical, radiological, and patient‐reported outcomes.

**What are the primary limitations to success in this case?**

- The primary limitation of this study is its reliance on a single case report.

## INTRODUCTION

Dental implants have demonstrated high long‐term survival rates regardless of their timing of placement.^1^ Contemporary treatment objectives in implant dentistry extend beyond mere implant survival, with both clinicians and patients now seeking functional and aesthetically pleasing outcomes that mimic natural dentition. Notably, the appearance of peri‐implant soft tissues has emerged as a crucial measure of success in implant therapy.^2^, ^3^ Due to increased esthetic expectations, a minor apical shift of the mucosal margin or a deficient interproximal papilla may be deemed as treatment failures, particularly in the esthetic region.^4^

The presence of complete interproximal papillae has major importance in anterior esthetics and warrants consideration in implant‐supported restorations.^5^, ^6^ In the early 1990s, Tarnow, Wagner, and Fletcher conducted a study examining the correlation between the distance from the contact point to the interproximal bone crest and the presence of the interdental papilla. Their findings revealed a critical threshold; when the measurement from the contact point to the bone crest was inferior to 5 mm, the papilla was present almost a hundred percent of the time.^7^ However, the same group showed that the supracrestal soft tissue height between two adjacent implants ranged between 2 to 4 mm.^8^ Consequently, this 1 to 2 mm of deficiency could lead to an aesthetic shortfall. From that point forward, numerous studies have investigated papillae in the context of single‐tooth implant treatment.^9^, ^10^, ^11^, ^12^, ^13^ Complete papilla fill was revealed to have an incidence of 58% between an implant‐supported restoration and a natural tooth.^11^ Particularly, the distal papilla around tooth‐bound implant‐supported restorations in maxillary central incisor sites exhibited an atrophied appearance after tissue maturation following the delivery of the final prosthesis.^13^

Generally, papilla height depends on the periodontal phenotype,^14^, ^15^ the distance between the contact point and the proximal bone crest,^10^, ^11^ and the distance between the implant and the adjacent tooth.^16^ According to Choquet et al., a complete papilla fill is expected when the distance from the contact point to the interproximal bone level is inferior to 5 mm.^11^ Subsequent research revealed that, for single‐tooth implants, the presence of papillae is determined by the interproximal bone level of the neighboring natural tooth rather than the bone level adjacent to the implant.^9^, ^12^ Particularly in cases involving maxillary central incisors replaced by implants, the atrophy of the distal papilla can be attributed to post‐extraction dimensional changes, the position of the CEJ on the mesial aspect of maxillary lateral incisors, and insufficient surgical or prosthetic management.^13^

The preservation and reconstruction of the interproximal papillae represent a challenge within the domain of implant dentistry. Diverse surgical and prosthetic methodologies have been proposed to address these challenges, with a focus on the preservation, restoration, and regeneration of the interproximal papillae adjacent to implants. These encompass papilla preservation procedures ^17^, ^18^ and hard and soft tissue augmentation techniques.^19^, ^20^, ^21^, ^22^, ^23^ For instance, Nemcovsky et al. introduced a papilla‐preservation technique during the second‐stage surgery, involving two vertical incisions to preserve the papillae of adjacent teeth and splitting the flap into mesial and distal parts, which are sutured to the anatomical papillae around the healing abutment.^24^ A decade later, Giordano et al. published a modification of the roll technique performed at the implant‐abutment connection, which consists of the preservation of the interproximal papilla using two vertical incisions and augmenting the buccal soft tissues with a pedicled graft derived from the de‐epithelization of the occlusal gingiva.^17^ Furthermore, Hürzeler et al. elaborated on a refined version of this technique, involving a tunneling approach toward adjacent teeth and employing microsurgical instruments to release the surgical papillae, followed by coronal positioning facilitated by double‐crossed sutures.^18^ On the other hand, hard tissue augmentation techniques could be performed to improve the bony architecture and the integrity of the bone peak, which is a crucial factor for interproximal papilla formation. Urban et al. described a multidisciplinary treatment approach involving a 4‐mm vertical bone augmentation procedure of the edentulous area, followed by soft tissue grafting at the re‐entry procedure. Although most papillae were regenerated, a minor discrepancy remained, necessitating the creation of a papilla‐supporting abutment.^20^ Additionally, Stefanini et al. showed a soft tissue augmentation approach in a case where the bone‐peak integrity was absent, using a modification of Zucchelli's connective tissue platform technique.^25^ Following submerged healing and implant‐abutment connection, papillae regrowth was successful thanks to prosthetic conditioning of the augmented soft tissues.^26^ Nevertheless, the outcomes of these procedures have proven to be unpredictable and there is currently no published evidenced‐based decision tree to guide clinicians with the most indicated treatment approach.

The objective of this case report is to showcase a soft tissue augmentation technique, performed at the time of implant placement, with the goal of enhancing interproximal papilla reconstruction following bone augmentation procedures.

## MATERIALS AND METHODS AND RESULTS

### Preoperative assessment

**FIGURE 1:**
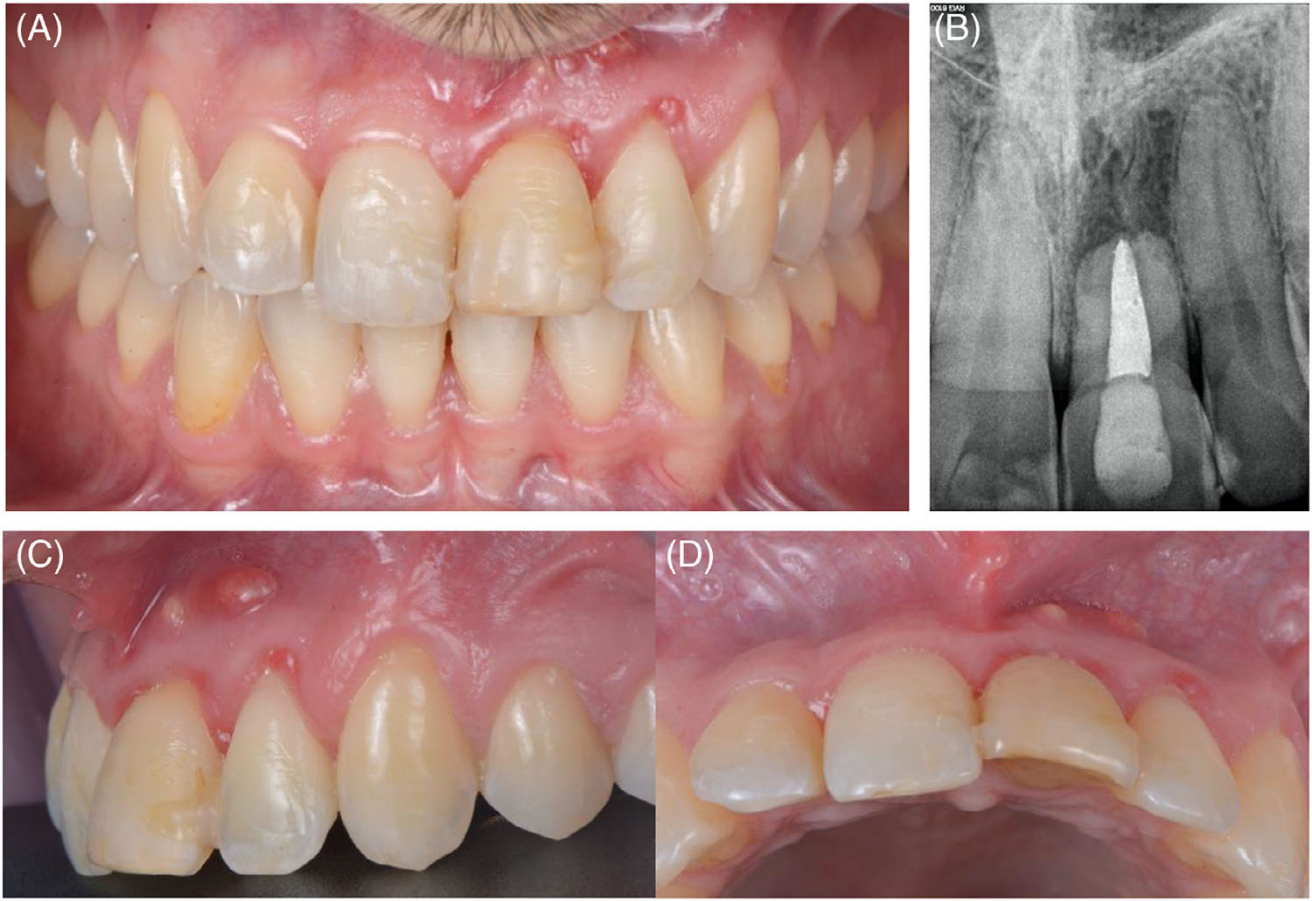
Initial case presentation. Frontal view of the patient´s occlusion (A). Periapical radiograph (B). Baseline lateral view (C). Baseline occlusal view (D).

A 28‐year‐old male patient was referred to a private practice (Blasi Dental Clinic Barcelona) in March 2022 for treatment. His chief complaint was the appearance of a fistula and discoloration of the upper left central incisor (Figure 1). Anamnesis showed a history of trauma in the anterior region. The patient gave written informed consent to proceed with the treatment.

### Clinical measurements

A comprehensive clinical and radiographic examination was conducted, including a periodontal assessment. The clinical exam revealed the presence of gingival inflammation, generalized gingival recessions, and class III mobility of the upper left central incisor. In addition, the patient presented a thin‐scalloped phenotype. Upon probing, a buccal bone dehiscence of 5 mm in length and 4 mm in width was detected.^27^

### Radiographic assessment

The radiographic exam showed an endodontically treated upper left central incisor with a periapical image and external root resorption. The patient was enrolled in a biofilm control program and received oral hygiene instructions. At the six‐week re‐evaluation, he showed a reduction in inflammation and improved plaque control.

The upper left central incisor was assessed to have a hopeless prognosis and was scheduled for extraction.^28^

### Surgical technique

**FIGURE 2:**
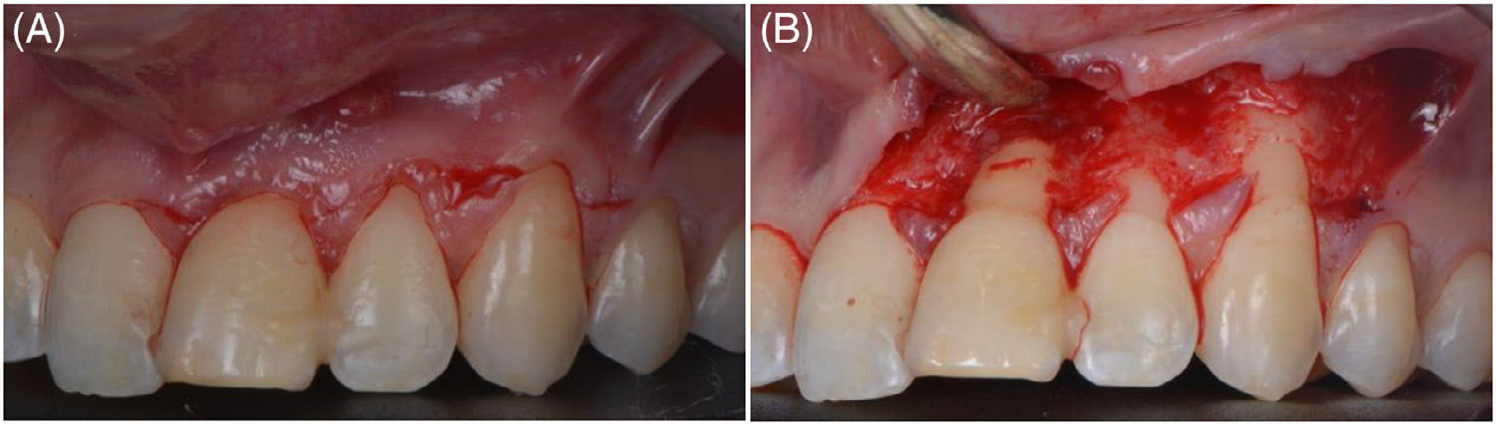
Incision and flap design. Oblique incisions (A). Split‐full‐split flap elevation (B).

**FIGURE 3:**
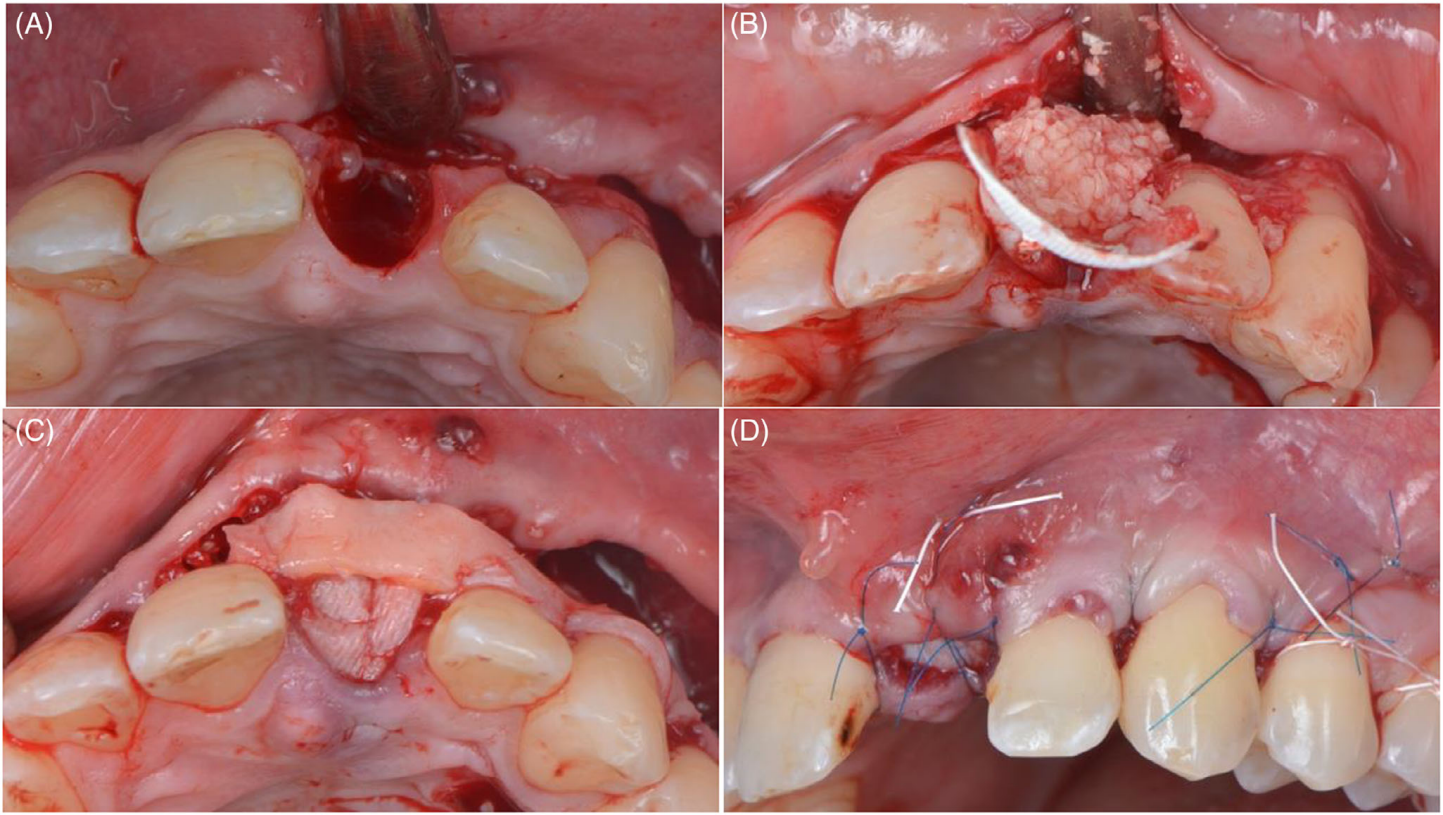
Hard and soft tissue augmentation. Extraction of the 2.1‐ note the presence of the dehiscence (A). Guided bone regeneration using a cross‐linked membrane and allograft bone particles (B). Suturing the connective tissue graft at the level of 2.1, 2.2, and 2.3 (C). Suturing of the flap (D).

To enhance mucogingival conditions of the adjacent teeth, a combination of root coverage and bone augmentation surgery was undertaken. Following buccal and palatal local anesthetic infiltration using 4% Articaine with 1:100000 epinephrine (Ultracain DS‐forte, Aventis Pharma, Frankfurt, Germany), oblique incisions were made from the upper right central incisor to the upper left canine and a vertical incision distal to the canine, adhering to coronally advanced flap principles ^29^ (Figure 2). A split full‐split flap was raised and the upper left central incisor was extracted atraumatically. The papillae were de‐epithelialized. The socket, exhibiting a 5 mm buccal bone dehiscence, underwent guided bone regeneration with freeze‐dried bone allograft (FDBA) particles (MinerOss Cortical, BioHorizons Camlog, Basel, Switzerland) and a cross‐linked collagen membrane (Ossix Plus, Datum Dental Ltd., Telrad, Israel). The membrane was fixed with horizontal mattress periosteal resorbable sutures (7‐0 Polyglactin 910, Vicryl, Ethicon, Johnson & Johnson, New Brunswick, NJ, USA).^30^ A connective tissue graft of 30 mm in length, 5 mm in width, and 1.5 mm in thickness was harvested from the palate after intra‐oral de‐epithelization using a wheel‐shaped diamond bur. The soft tissue graft was sutured to the de‐epithelized anatomical papillae, at the level of the cementoenamel junction, also extending to the upper right central incisor using 7‐0 polyglactin sutures. The flap was then advanced and sutured with interrupted and sling sutures using non‐resorbable sutures (6‐0 Polypropylene, Prolene, Ethicon, Johnson & Johnson, New Brunswick, NJ, USA and 4‐0 polytetrafluoroethylene PTFE, Hu‐Friedy Group, Chicago, IL, USA), leaving the bone augmentation area to heal by secondary intention (Figure 3).

### Postoperative indications

Antibiotics (amoxicillin 500 mg, 8 hours) and analgesics (ibuprofen 600 mg, 8 hours) were prescribed along with an antimicrobial rinse (0.12% chlorhexidine, twice daily for 2 weeks). Oral hygiene was discontinued in the surgical area and non‐mechanical plaque control was recommended for 2 weeks.

### Follow‐up appointments

The healing was uneventful and sutures were removed after 14 days.

### Re‐entry procedure

**FIGURE 4:**
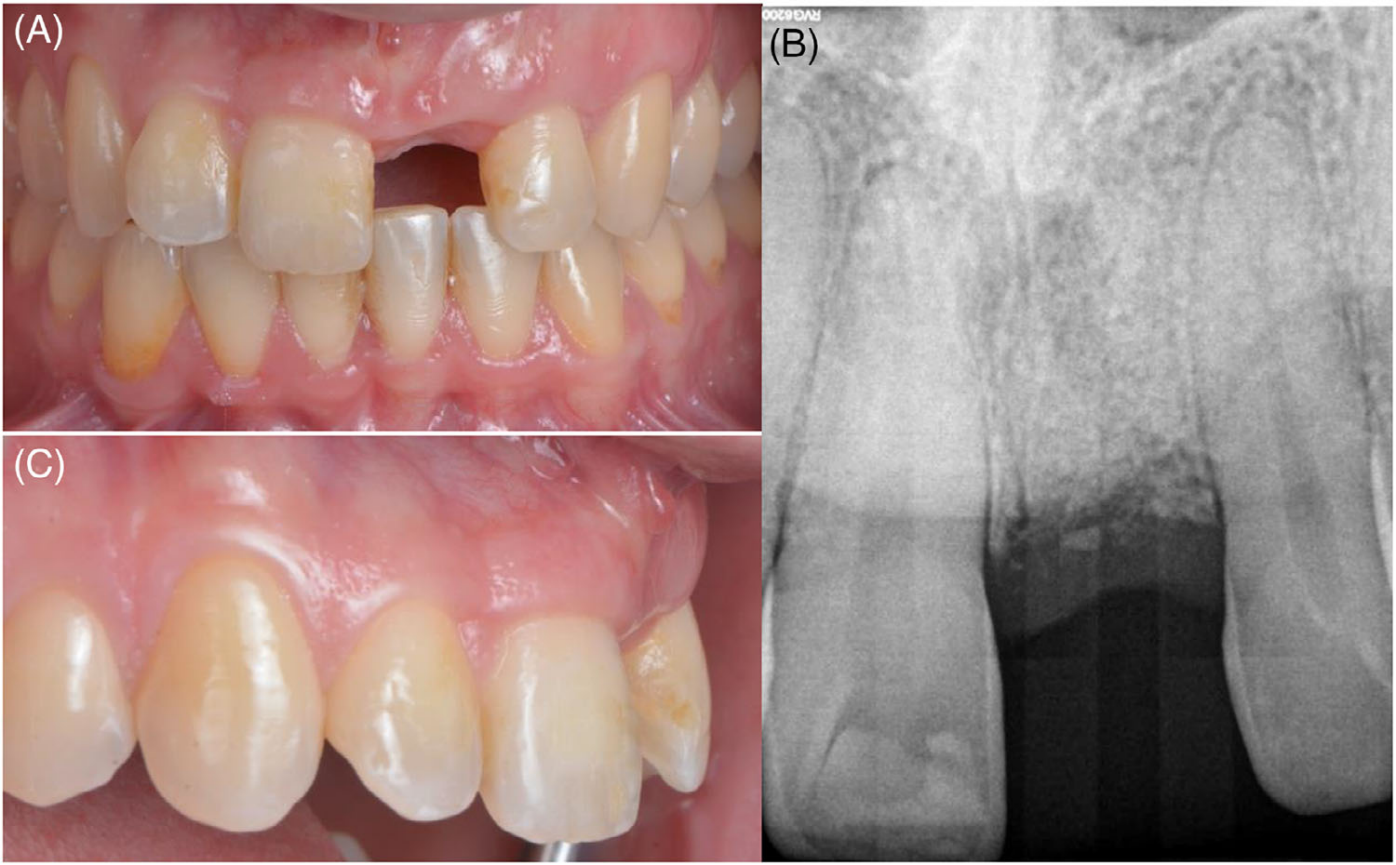
Healing at 4 months. Frontal view (A). Lateral view (C). Periapical radiograph (B).

**FIGURE 5:**
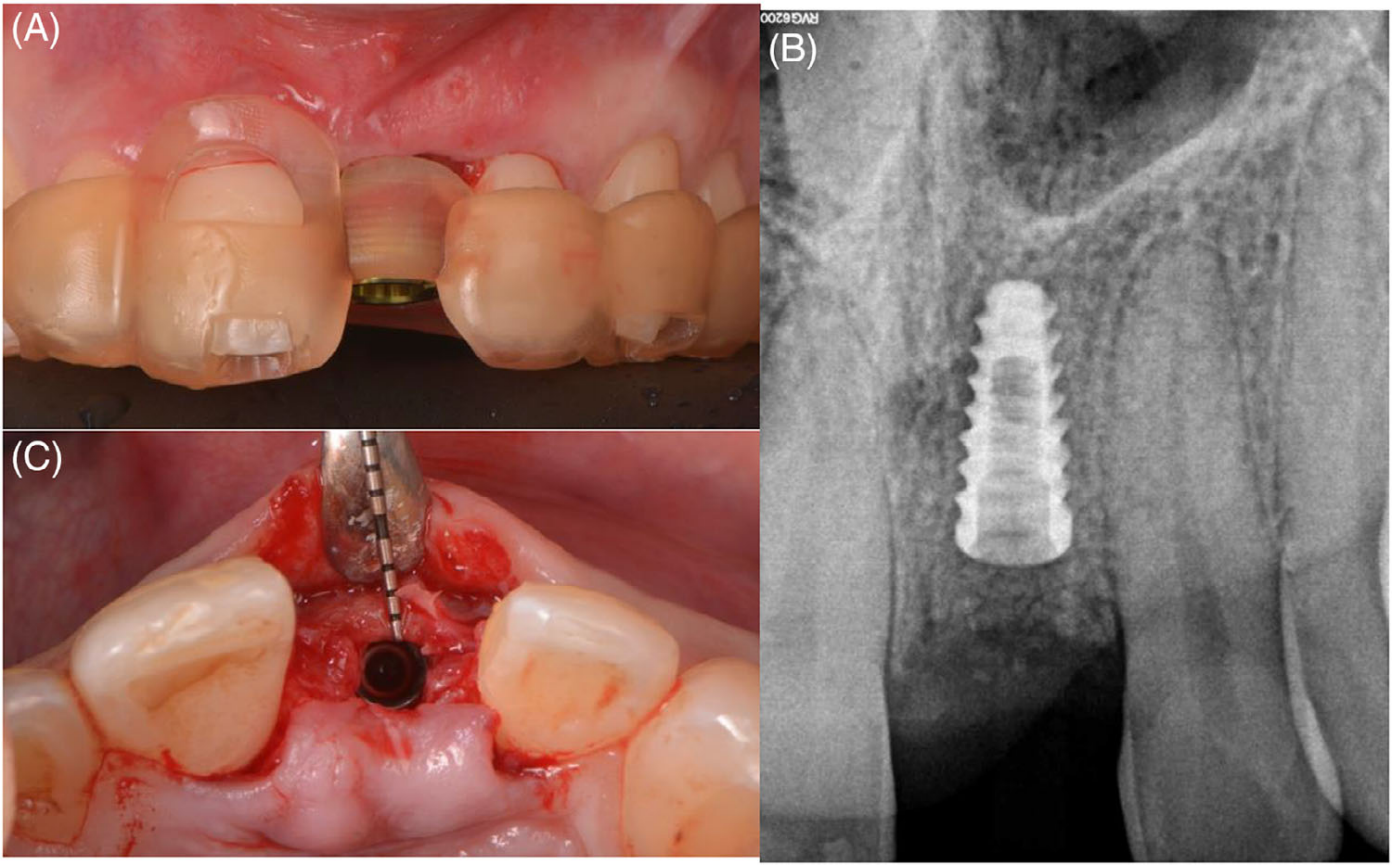
Re‐entry. Implant placement using a fully guided protocol (A). Critical buccal bone thickness present (C). Periapical radiograph post‐implant placement (B).

**FIGURE 6:**
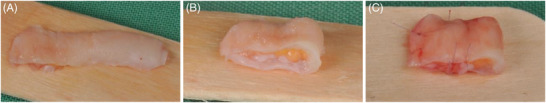
Connective tissue graft harvesting. Deepithelialized free gingival graft (A). Folding of the graft in two (B). Suturing with 6‐0 resorbable sutures (C).

**FIGURE 7:**
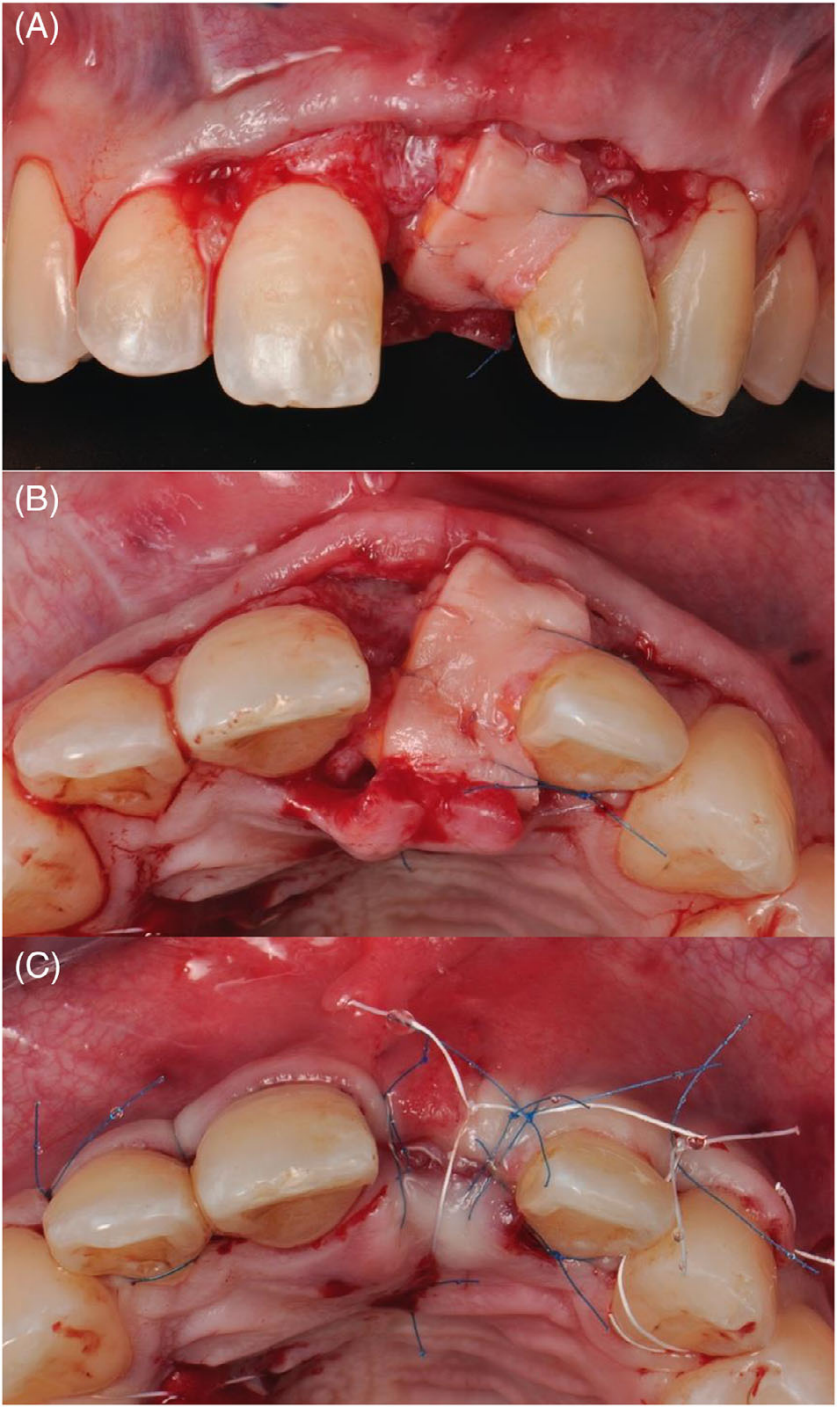
Connective tissue graft and flap suturing. The distally anchored connective tissue graft platform—frontal view (A). The distally anchored connective tissue graft platform—occlusal view (B). Flap sutured, achieving primary closure (C).

After 4 months, the re‐entry procedure was performed (Figure 4). A crestal incision was followed by a full‐thickness flap. A bone‐level implant of 3.8×10.5 mm (BioHorizons Tapered Plus, BioHorizons Camlog, Basel, Switzerland) was placed using a fully guided protocol and submerged healing was allowed. A buccal bone thickness of 2 mm was observed (Figure 5). To further enhance the soft tissue volume, another connective tissue graft was performed at the time of implant placement. Following intra‐oral de‐epithelization using a wheel‐shaped diamond bur, a connective tissue graft of 25 mm in length, 6 mm in width, and 1.5 mm in thickness was harvested from the palate. The graft was folded in two and sutured together with 7‐0 polyglactin sutures to increase its thickness. The autogenous graft was then positioned on the occlusal and buccal surfaces and distally anchored around the left lateral incisor using sling suture (i.e. Distally anchored connective tissue platform) (Figure 6). To achieve this, a 6‐0 polypropylene suture was employed initially to penetrate the graft in the disto‐palatal aspect, traversing through the contact point between the left lateral and canine teeth to access the buccal side. Subsequently, a second suture was performed in the disto‐buccal aspect of the graft, followed by a return to the palatal side to tie a surgeon's knot. Oblique incisions were performed on the distal papillae of the right central and the left lateral incisors, and the latter were further de‐epithelized. The flap was released and sutured to achieve primary closure with interrupted and sling sutures using 6‐0 polypropylene and 4‐0 PTFE (Figure 7).

### Postoperative indications

Postoperative care included antibiotics, analgesics, and an antimicrobial rinse for 2 weeks, with a brief cessation of oral hygiene in the surgical area.

### Follow‐up appointments

The healing was uneventful and the sutures were removed after 14 days.

### Prosthetic phase

**FIGURE 8:**
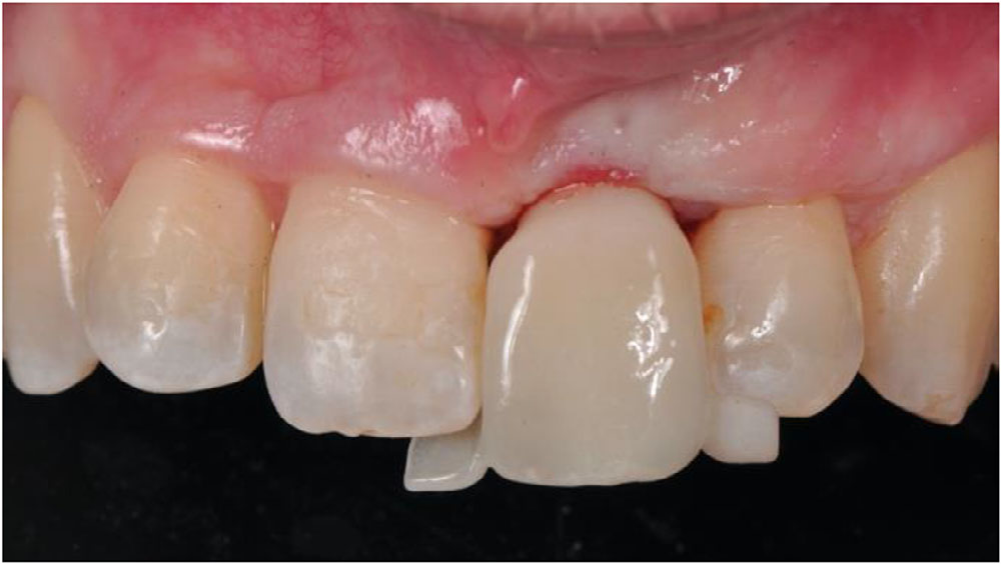
Non‐invasive second stage using the provisional restoration.

**FIGURE 9:**
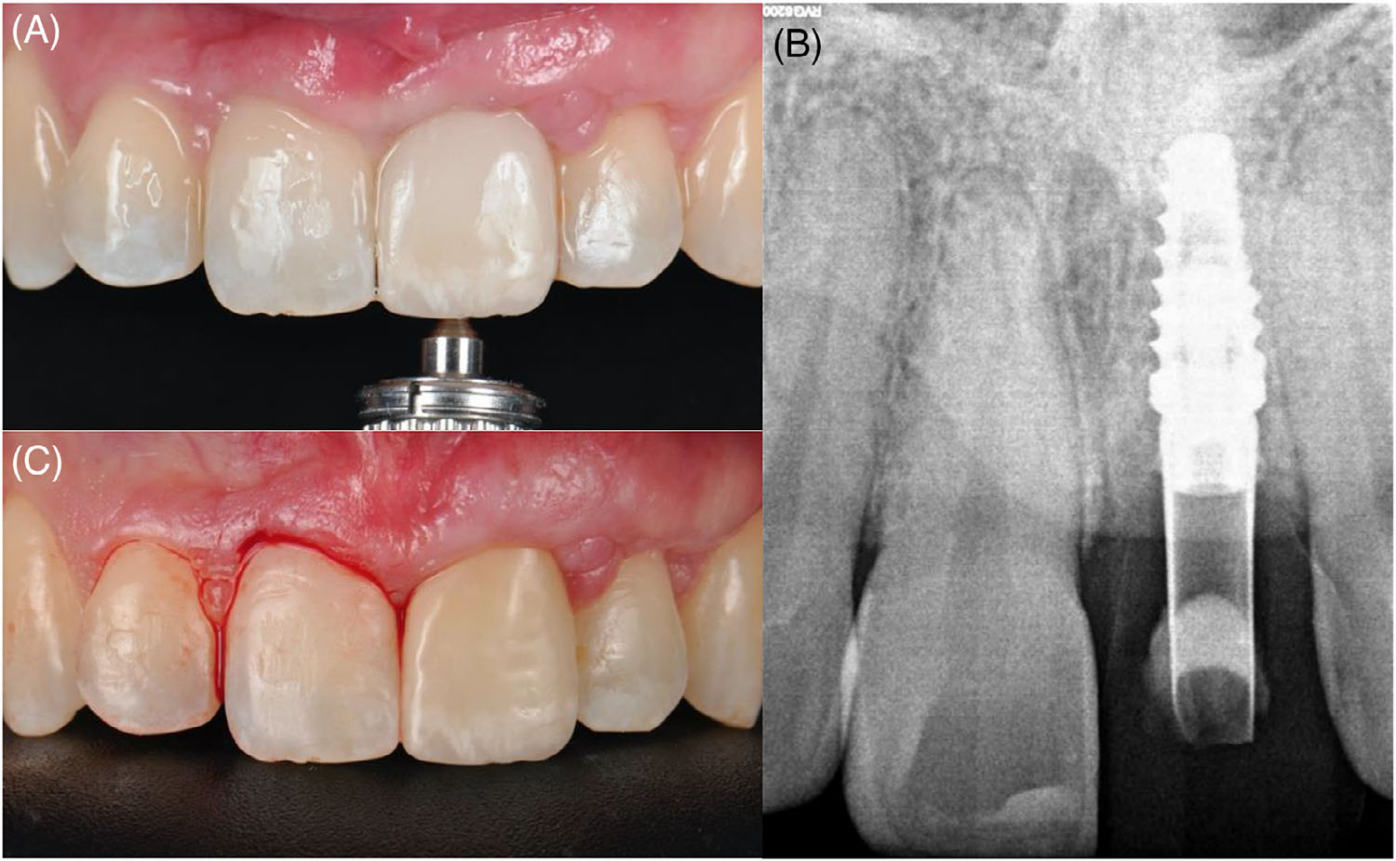
Implant loading. Frontal view, 2 weeks after provisional connection (A). Six weeks after provisional—some gingivectomy in the upper right central (C). Periapical radiograph (B).

**FIGURE 10:**
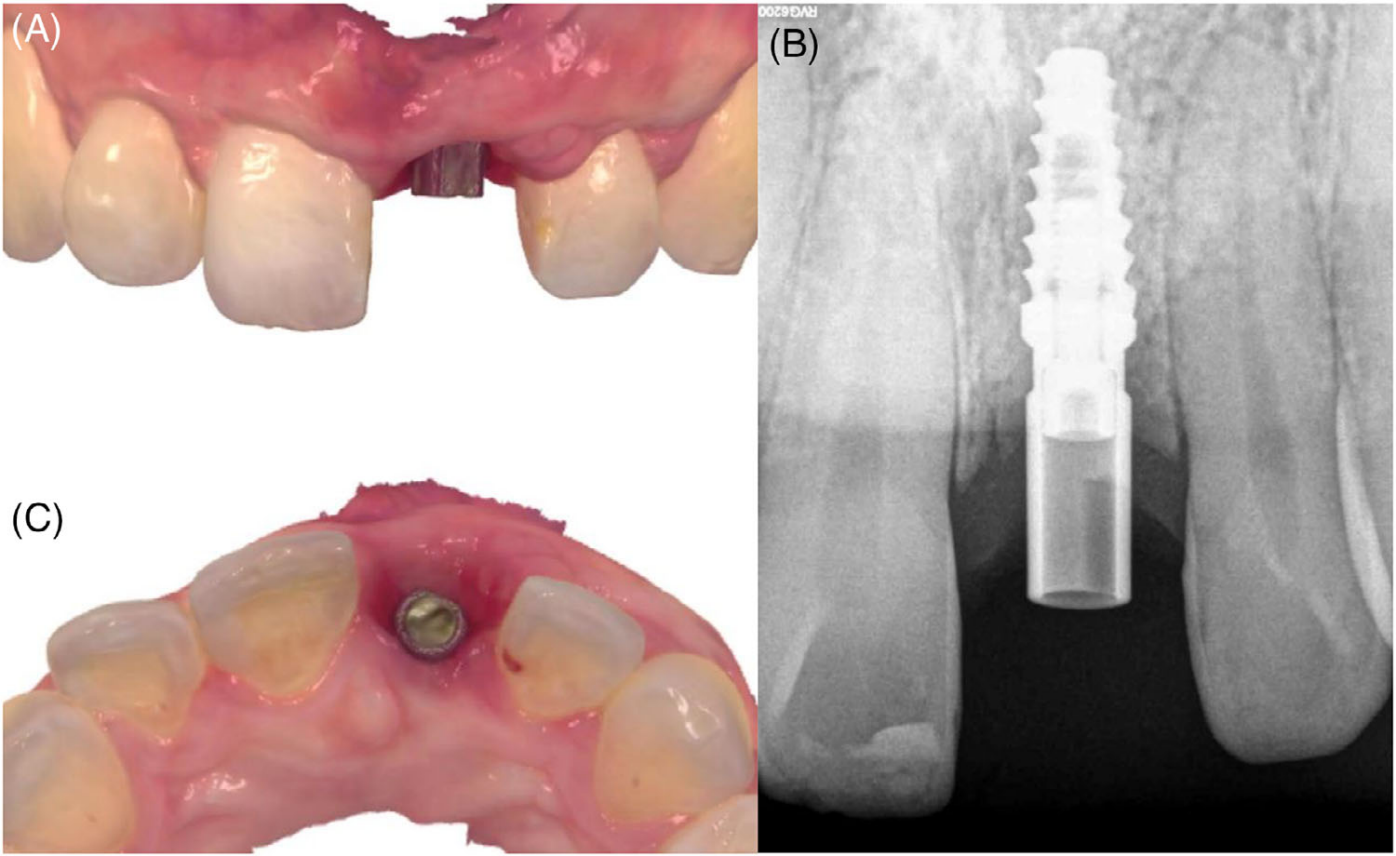
Digital impression. Frontal view of the intra‐oral scan (A). Occlusal view of the intra‐oral scan (C). Periapical radiograph (B).

**FIGURE 11:**
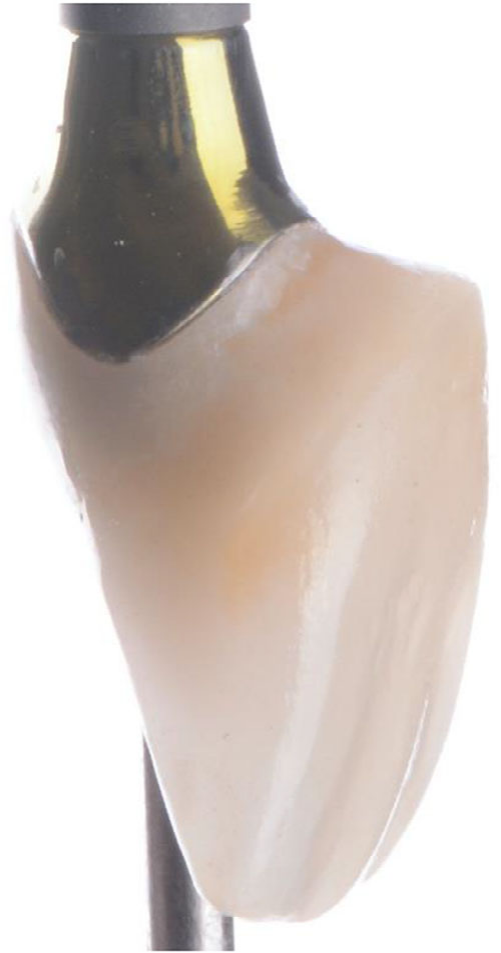
Definitive crown.

After 2 months, a Maryland provisional restoration was fabricated with an ovoid pontic design, and incremental pressure was applied to the soft tissues adding flow composite weekly. The goal was to use a non‐invasive method to reach the cover screw (Figure 8). This was achieved in the fourth week when the provisional restoration was connected to the implant. In this session, the critical contour was modified to permit a coronal migration of the mucosal margin. After 6 weeks of the implant loading, the desired mucosal margin level was reached on the upper left central, and a small gingivectomy was performed on the contralateral incisor to level the gingival margin levels (Figure 9). 3 weeks later, final digital impressions were taken (3Shape Trios, Copenhagen, Denmark) (Figure 10), and a screw‐retained zirconia crown with an anatomical abutment was delivered (Figure 11).

### Outcomes

**FIGURE 12:**
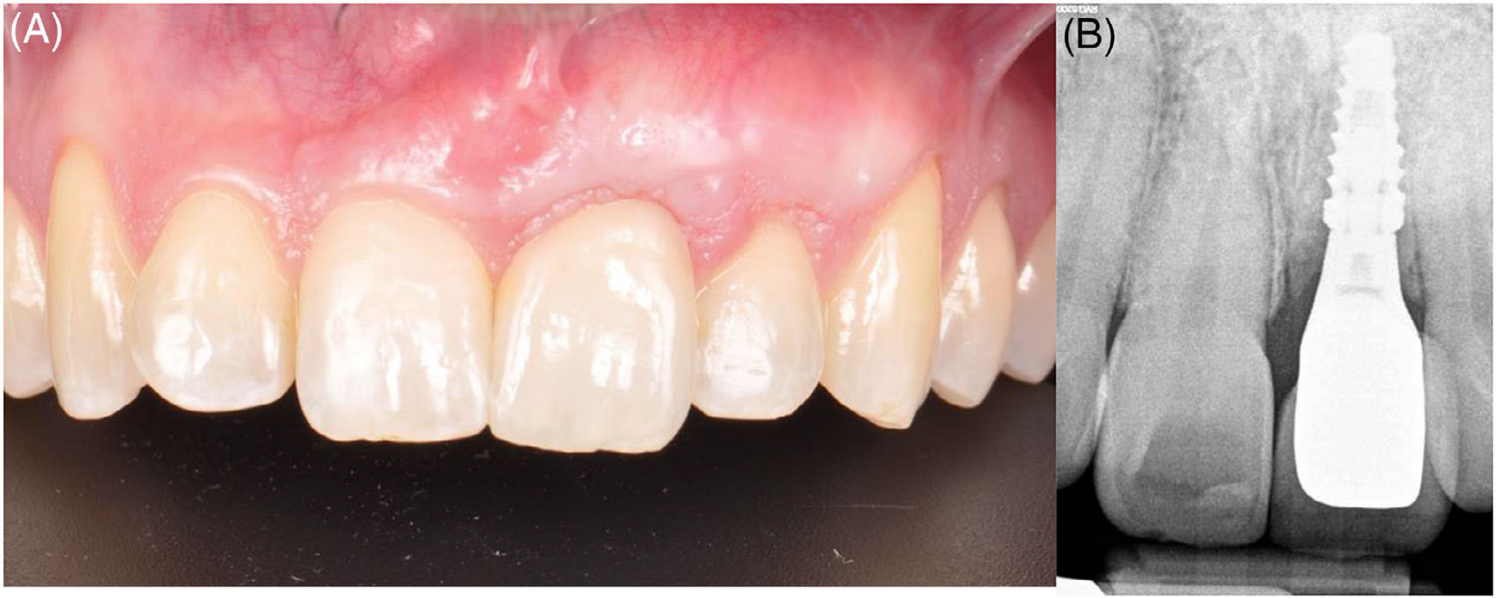
Final follow‐up. Frontal view (A). Periapical radiograph (B).

The achieved clinical outcomes were favorable, revealing stable mucosal margin levels, sufficient keratinized tissue, substantial mucosal thickness, and most importantly complete papilla fill (Figure 12). The patient expressed satisfaction with the achieved results.

## DISCUSSION

The present case report illustrates the successful management of a compromised socket in the maxillary anterior area through a combination of hard and soft tissue regeneration techniques, leading to a final natural implant‐supported restoration. The case demonstrated favorable esthetic outcomes in both pink and white aesthetics.^31^ Notably, complete mesial and distal papillae fill were achieved.

The distally anchored connective tissue platform technique featured in this report enables the augmentation of soft tissues in both horizontal and vertical dimensions, specifically focusing on facilitating distal papilla formation. The rationale behind this technique lies in peri‐implant phenotype modification promoting enhanced papilla formation. Indeed, a systematic review by Bienz et al. demonstrated superior papilla scores associated with increased soft tissue thickness and a heightened likelihood of papilla presence in thicker phenotypes.^32^ Moreover, anchoring the connective tissue graft distally using sling sutures permits the preservation of the distal papilla between the maxillary central and lateral incisors, a region often prone to atrophy following final prosthesis delivery.^13^

In this instance, two soft tissue augmentation interventions were conducted: one during tooth extraction and ridge preservation, and another during implant placement. These interventions play crucial roles in achieving optimal papilla fill. Additionally, the preservation of papillae in this case can be attributed to several other factors, such as the meticulous management of the compromised socket through alveolar ridge reconstruction on the day of tooth extraction. This approach maximized the stability of the alveolar ridge architecture, thereby contributing to the preservation of an adequate papillary anatomy. Furthermore, effective bone regeneration obviated the need for additional bone augmentation at the time of implant placement, mitigating potential soft tissue alterations and scar tissue formation.^33^ In addition, opting for a secondary intention healing played a crucial role in preserving the width of keratinized tissue, as evidenced in a randomized clinical trial by Barone et al., which demonstrated a gain of 1.8 mm in the keratinized tissue width in the secondary intention healing group compared to a loss of 1.7 mm in the mucoperiosteal flap group.^34^ Furthermore, implant placement with a fully guided approach facilitated a prosthetically‐driven ideal 3D position, aiding in papillae formation post–guided bone regeneration. Literature underscores the importance of accurate implant placement, as inaccuracies may lead to distal papilla deficiency due to extensive peri‐implant bone remodeling or proximity to adjacent teeth.^35^ Finally, care was taken to perform the second‐stage procedure with the utmost minimally invasive measures to prevent trauma to the papillary areas and subsequent soft tissue contraction.^36^

This case report emphasizes the meticulous planning required to establish ideal hard and soft tissue architecture for future implant‐supported restorations. Informed diagnoses and decisions made prior to tooth extraction play a crucial role in ensuring the long‐term stability of both soft and hard tissues, thereby reducing the risk of biological and esthetic complications. Research indicates that dimensional changes occur following implant placement in healed ridges, potentially leading to instability of peri‐implant hard and soft tissues. Sites with thin buccal bone are particularly susceptible to significant changes that may compromise bone integrity and increase the likelihood of biological complications such as peri‐implant mucositis and peri‐implantitis.^37^ Indeed, a four‐year clinical study demonstrated that implants with residual buccal dehiscence defects were more prone to developing peri‐implantitis.^38^  Furthermore, soft tissue parameters, including soft tissue thickness, play a role in marginal bone level and peri‐implant mucosal margin stability.^39^, ^40^, ^41^ A systematic review concluded that implants placed with thicker peri‐implant soft tissues exhibit less radiographic marginal bone loss in the short term.^42^ Among esthetic complications, peri‐implant soft tissue dehiscences (PSTD) are prevalent, with some studies reporting a prevalence of 57–64%.^43^, ^44^ Among the predisposing factors are the lack of keratinized mucosa ^45^, ^46^, ^47^ and implant malposition, particularly bucco‐lingual implant positioning.^48^, ^49^ These findings underscore the importance of careful treatment planning to minimize the risk of biological and esthetic complications and optimize long‐term outcomes in implant dentistry.

This case report demonstrates favorable clinical, radiographic, and patient‐reported outcomes. However, the described technique may pose certain limitations. Firstly, it relies on harvesting autogenous soft tissue from the palate, a procedure associated with higher postoperative morbidity than soft tissue substitutes. Specifically, pain ranks as the most common postoperative complication following palatal harvesting, with some patients reporting discomfort associated with the procedure even a decade after its completion.^50^ Moreover, performing multiple soft tissue grafts also entails a higher financial cost for the patient. This case report demonstrates successful papilla fill by utilizing the distally anchored connective tissue platform in a single‐tooth implant adjacent to teeth with no attachment loss, facilitating the preservation of papillary architecture. Existing evidence suggests that complete papilla fill typically depends on the interproximal attachment of adjacent teeth.^9^, ^12^, ^51^ However, without further evidence, it is challenging to speculate whether this technique would yield successful papilla fill in patients with stable periodontitis.

## CONCLUSION

The distally anchored connective tissue graft platform, performed at the time of implant placement is a viable and promising soft tissue augmentation technique that yields favorable clinical and esthetic outcomes. Prospective clinical studies and controlled trials are further needed to assess the efficacy and predictability of this technique.

## AUTHOR CONTRIBUTIONS

GB, LA, and AB prepared the study protocol; GB performed the surgeries, and AB delivered the prosthetic treatment; GB, LA, and AB led the writing; GB, LA, and AB revised the draft manuscript.

## CONFLICT OF INTEREST STATEMENT

No conflicts to disclose.
